# Development of a Transcultural Social Ethical and Integrated Care Model, for Dependent and Older People Populations at Risk of Exclusion in the Mediterranean Sea Basin (TEC-MED): A Research Protocol

**DOI:** 10.5334/ijic.7782

**Published:** 2024-12-19

**Authors:** Ana María Porcel-Gálvez, Elena Fernández-García, Soledad Vázquez-Santiago, Sergio Barrientos-Trigo, María Dolores Mateos-Garcia, Mercedes Bueno-Ferrán, Jalila El Ati, Hajer Aounallah-Skhiri, Marta Lima-Serrano

**Affiliations:** 1 Departamento de Enfermería, Facultad de Enfermería, Fisioterapia y Podología, Universidad de Sevilla, Spain; 2 Research Group CTS-1050 “Complex Care, Chronicity, and Health Outcomes”, Instituto de Biomedicina de Sevilla, Spain; 3 IBiS/Hospital Universitario Virgen del Rocío/CSIC/Universidad de Sevilla, Seville, Spain; 4 Research Group CTS-1141 “Clinical Research Applied to Care and New Care Paradigms (ICCAPA)”, Instituto de Biomedicina de Sevilla, IBiS/Hospital Universitario Virgen del Rocío/CSIC/Universidad de Sevilla, Seville, Spain; 5 Research Group CTS-1050 “Complex Care, Chronicity, and Health Outcomes”, Instituto de Biomedicina de Sevilla, IBiS/Hospital Universitario Virgen del Rocío/CSIC/Universidad de Sevilla, Seville, Spain; 6 Research Group CTS-1050 “Clinical Research Applied to Care and New Care Paradigms (ICCAPA)”, Instituto de Biomedicina de Sevilla, IBiS/Hospital Universitario Virgen del Rocío/CSIC/Universidad de Sevilla, Seville, Spain; 7 INNTA (National Institute of Nutrition and Food Technology), Tunisia; 8 SURVEN (Nutrition Surveillance and Epidemiology in Tunisia) Research Laboratory, Tunis, Tunisia; 9 University Tunis El Manar, Tunis, Tunisia; 10 INSP (National Institute of Health), Faculty of Medicine of Tunis, Tunisia; 11 Research Group CTS-969 “Care Innovation and Social Determinants of Health”, Instituto de Biomedicina de Sevilla, IBiS/Hospital Universitario Virgen del Rocío/CSIC/Universidad de Sevilla, Seville, Spain

**Keywords:** dependency, integrated care, older people, risk of exclusion, transcultural, Atención integrada, Dependencia, Personas mayores, Riesgo de exclusión, Transcultural

## Abstract

**Background:**

Higher life expectancy has produced a higher older people porpulation, not necessarily with a consistent quality of life, showing a high rate of vulnerability and dependence. The current social and health crisis situation has highlighted the need to create new integrated models of care that could be translated into social and health policies.

**Objective:**

The present study aims to develop, test, and validate an innovative integrated care model for older people with dependence and at risk of social exclusion and their caregivers.

**Methods:**

The TEC-MED project participants are nine project partners and six associated partners, with geographic coverage from six countries in the Mediterranean basin, Spain, Tunisia, Italy, Lebanon, Egypt, and Greece. Project coordination will take place at three different levels, macro, meso and micro, through six work packages. The pilot phase uses qualitative-quantitative method to assess the impact of the TEC-MED project, it is expected to train 36 Training Agents, six per country, who apply to a total of 28,200 people (mainly elders and their caregivers).

**Conclusion:**

The study proposes a new social integrated care organizational model focused on the integration of social and health care, comprising the governance model; the organization structure and the skills profiles for the caring personnel.

## Abstract (es)

**Antecedentes:**

La mayor esperanza de vida ha generado un incremento en la población de personas mayores, sin embargo, no necesariamente se ha traducido en una mejora constante de la calidad de vida, evidenciándose una alta tasa de vulnerabilidad y dependencia. La actual crisis social y sanitaria ha resaltado la necesidad de crear nuevos modelos integrados de atención que puedan traducirse en políticas sociales y de salud.

**Objetivo:**

El presente estudio tiene como objetivo desarrollar, probar y validar un modelo innovador de atención integrada para personas mayores con dependencia y/o en riesgo de exclusión social, así como para sus personas cuidadoras.

**Métodos:**

Los participantes del proyecto TEC-MED son nueve socios del proyecto y seis asociados, con cobertura geográfica en seis países de la cuenca del Mediterráneo: España, Túnez, Italia, Líbano, Egipto y Grecia. La coordinación del proyecto se llevará a cabo en tres niveles diferentes, macro, meso y micro, y a través de seis paquetes de trabajo. La fase piloto utiliza un método cualitativo-cuantitativo para evaluar el impacto del proyecto TEC-MED. Se espera capacitar a 36 Agentes de Formación, seis por país, quienes aplicarán el proyecto a un total de 28,200 personas (principalmente personas mayores y sus personas cuidadoras.).

**Conclusión:**

El estudio propone un nuevo modelo organizativo de atención social integrada, centrado en la integración de la atención social y sanitaria, que comprende el modelo de gobernanza, la estructura organizativa y los perfiles de competencias del personal de cuidado.

## Introduction

Currently, 200 million people, 12% of the world population, have reached or exceeded the life expectancy of health (60 years) [1]. However, increased life expectancy is not associated with higher quality of life. Indeed, older adults often become more vulnerable as they age, encountering a greater risk of social exclusion or dependence, which implies greater spending on resources and greater use of community resources. Social health institutions and policies must face these issues. A main goal of the 2030 Agenda for Sustainable Development is to improve health and education and reduce inequalities, pursuing the 17 Sustainable Development Goals (SDGs) [2].

In this context, social determinants of health must be considered as a framework, which are the social conditions in which people work, live, and age that negatively affect health outcomes and lead to health inequalities, economic income is a very important factor [3]. Another relevant framework is integrated care, which is a healthcare delivery approach that focusses on coordinating and providing comprehensive and seamless care to individuals in different healthcare settings and disciplines of healthcare. The goal of integrated care is to improve patient outcomes, improve patient experiences, and reduce healthcare costs by breaking down silos and promoting collaboration between healthcare providers, such as primary care physicians, specialists, nurses, social workers, mental health professionals, and other professionals involved in patient care [4]. This reflects the need for the coordination and real integration of social and health care and services to increase health and well-being in the population, especially the older people, by promoting the capacities and competencies of care providers [3]. Moreover, integrating social and health care services could reduce bureaucracy and improve care [5]. Experience with the integration of social and health care can be found in the literature. For example, Finland recently implemented a reform to integrate these [6]. However, other contexts, such as in the countries of the Mediterranean basin, have not yet incorporated social and health policies.

Until now, there is no comprehensive care model that encompasses both health-related and social aspects in this context. When a social model is developed, culture must be considered [7,8,9]. Culture, which incorporates values, beliefs, ways of living, and traditions that are transmitted from generation to generation, configures behaviors and ways of being and acting [10]. Universal social and health care needs (common to all cultures) and diverse aspects (specific care obtained in each culture) must be considered [11,12]. People from different cultures can inform and guide professionals to receive the type of care they need. Social and health workers must discover and acquire knowledge of the world and, using it, make appropriate decisions to provide care that is consistent with culturally marked care [11].

As universal ethical value, dignity, active aging, and long-term care must be guaranteed [13]. Autonomy must be taken into account as supporting the right of a person to determine his or her own destiny, beneficence as a way of doing good, and justice as a way of sharing benefits and burdens based on fairness and equality, such as in the provision of privacy and confidentiality. Overlooking any of these ethical principles brings the risk of neglect or abuse [13,14].

Finally, the framework of the Patient-Centered Culturally Sensitive Health Care Model is useful for explaining the link between the provision of patient-centered, culturally sensitive health care and the health behaviors and outcomes of the patients who experience this care [15,16].

Taking into account these frameworks and assumptions, the TEC-MED research project addresses the challenges of older populations with dependence and at risk of social exclusion, by developing a transcultural, ethical, and social care model for dependent populations in the Mediterranean basin to be implemented in six countries (Spain, Italy, Greece, Lebanon, Egypt, and Tunisia). The Mediterranean countries share cultural ties, similar challenges and needs. The goal of the TEC-MED project is to improve quality of life and reduce marginalization among the target population as well as to benefit their caregivers and to improve the quality of social and health services provided to them. By developing a common model supported by software applications, this project can help establish a benchmark model for social assistance in the Mediterranean region.

## Aim

This paper is aimed to present the TEC-MED project and its methodology.

The TEC-MED project objectives are to 1) develop the TEC-MED model, an innovative integrated transcultural social care model to improve daily operations in social enterprises and collaborate with public administration in the care of dependent older people who are at risk of social exclusion; 2) To test the TEC-MED model, through six case studies in which participates nine project partners and six associated partners, with a geographic coverage of six countries in the Mediterranean basin, namely Spain, Tunisia, Italy, Lebanon, Egypt, and Greece.

At the end of the TEC-MED project we expected to:

1. Built an intervention framework to improve the operations of social enterprises and collaboration with public administration to implement actions that improve the social care conditions of older people that have difficulty in economic and social integration.
2. Developed a software solution including an online platform for a cooperative partnership between public institutions and social care actors.
3. Validated the model through six pilot actions in participating countries implementing the action for testing with social enterprises, citizens, and public administrations.
4. Promoted usability by training professional caregivers in the model—the trained caregivers will be called training agents (TA). These TAs are professionals with specialized training in the socio-health field such as nurses, social workers, psychologists specially qualified and trained in the TEC-MED model to be able to care for dependent older people and at risk of social exclusion. These professionals will be trained in the TEC-MED model and will be responsible for training the rest of the participating entities in its use and implementation and joint training among social service people working in public authorities, associations, social-care service entities, professionals and technicians.
5. Use the results of the study to formulate a governance model, organizational structure, skilled profiles for social care personnel, software solutions that allows the exchange of information on country-based initiatives to facilitate the social inclusion of older people, and cross-country analysis for an effective comparison.

## Description of the methodology

### Target Population

**Figure 1:**
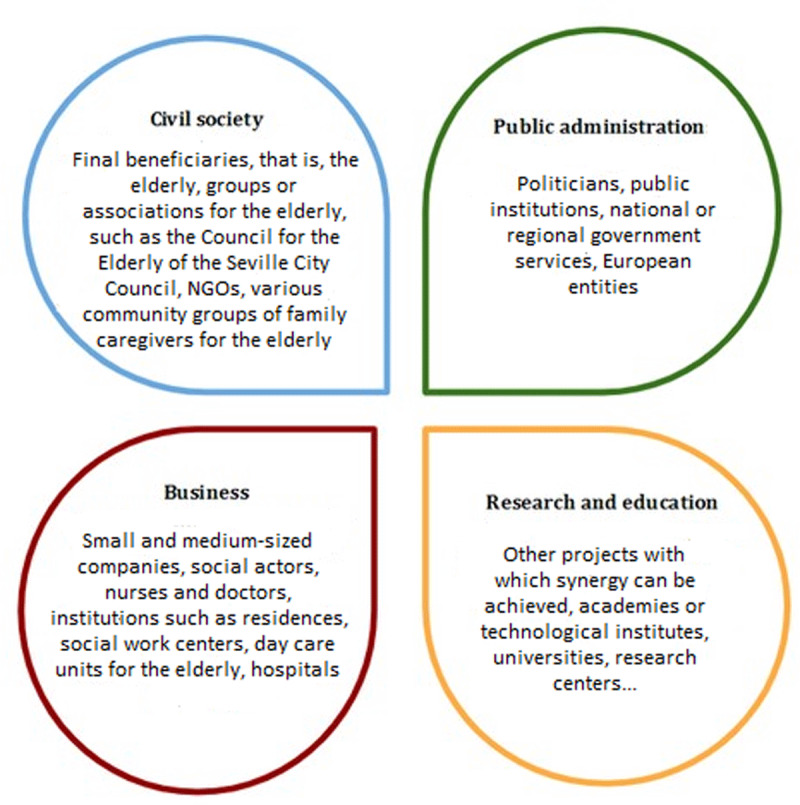
Quadruple helix describing the TEC-MED project.

The final beneficiaries of the TEC-MED project are people 60 years of age or older with dependencies and who are at risk of social exclusion. In addition, caregivers in their family are served by this project. The target group for the project is relevant stakeholders in the field of social care provided, such as policy makers, social care operators, nongovernmental organizations (NGOs), research institutes, and civil society organizations. classified using the quadruple helix (Figure 1). This model allows the shared characteristics of community actors to be determined, that is, determine the sector that an actor operates or represents [17,18]. The methodology seeks to involve all the agents needed to produce a social impact to improve social conditions and project performance. Both the final beneficiaries and the target groups of the project will be directly involved as key participants in the project. The needs of both the final beneficiaries and the target group are addressed in the TEC-MED model.

### Sample

In the pilot phase, new professional careers for the TA are expected to be developed. Six TAs are to be trained in each country to reach a total of 28,200 people considering the time of the pilot phase (4.700 in each country) mainly including final beneficiaries and caregivers. TAs are professionals with a specific profile, education, and experience for accessing the target population. There will be approximately a TA for every 784 patients per country in six countries; TAs will assess social and health needs, make a social diagnosis, and provide an intervention plan. Regarding the inclusion criteria, personnel with experience of working with older people, who have training related to social and health care, will be selected. The training programs that will be carried out will include training in specific aspects of the TEC-MED model, such as basic care for dependent older people or those at risk of social exclusion.

The rest of stakeholders (considering the quadruple helix) will be involved in the development (Work packages 3, 4 and 6), the implementation and the qualitative and quantitative evaluation of the model (Work packages 5), with the intention to make this more relevant and sustainable. These stakeholders include: the European Commission and its institutions, which represent the EU’s interests and enforce its laws; policymakers, responsible for creating or amending policies using research evidence; social care operators that provide services for the older people in need; social and healthcare providers including doctors, nurses, social workers as well as social care manager at social-health institutions; research institutions and universities that drive innovation in older people care; and, finally, citizens and community groups often volunteer in social-health care efforts.

### Logical Framework and Schedule

The TEC-MED project is expected to produce several innovative results for the territories involved over the 36 months of the project (2019–2022). The project coordination takes place at three different levels: 6 work packages (WPs), 17 activities, and 10 actions (6 project pilots, 3 knowledge exchange workshops, and 1 on-line sharing platform).

Each WP has a WP leader, and its action is subdivided into a series of activities. The WP leaders bear the ultimate responsibility for the timely completion of the activities in accordance with predefined quality standards. For this reason, the WP leaders must maintain close contact with all activity leaders to ensure smooth coordination between the Partners Projects (PPs). Involved partners are: Leader Beneficiary (University of Seville), PP1 (Magtel Operations), PP3 (VIDAVO), PP4 (Merimna), PP5 (National Institute of Nutrition and Food Technology), PP7 (Institute for Development, Research, Defense, and Applied Attention (IDRAAC)), PP8 (Academy of Scientific Research and Technology), PP9 (SEKEM Development Foundation), PP10 (Uni Camilus), from Spain, Greece, Tunisia, Lebanon, Egypt and Italy, respectively.

**Figure 2:**
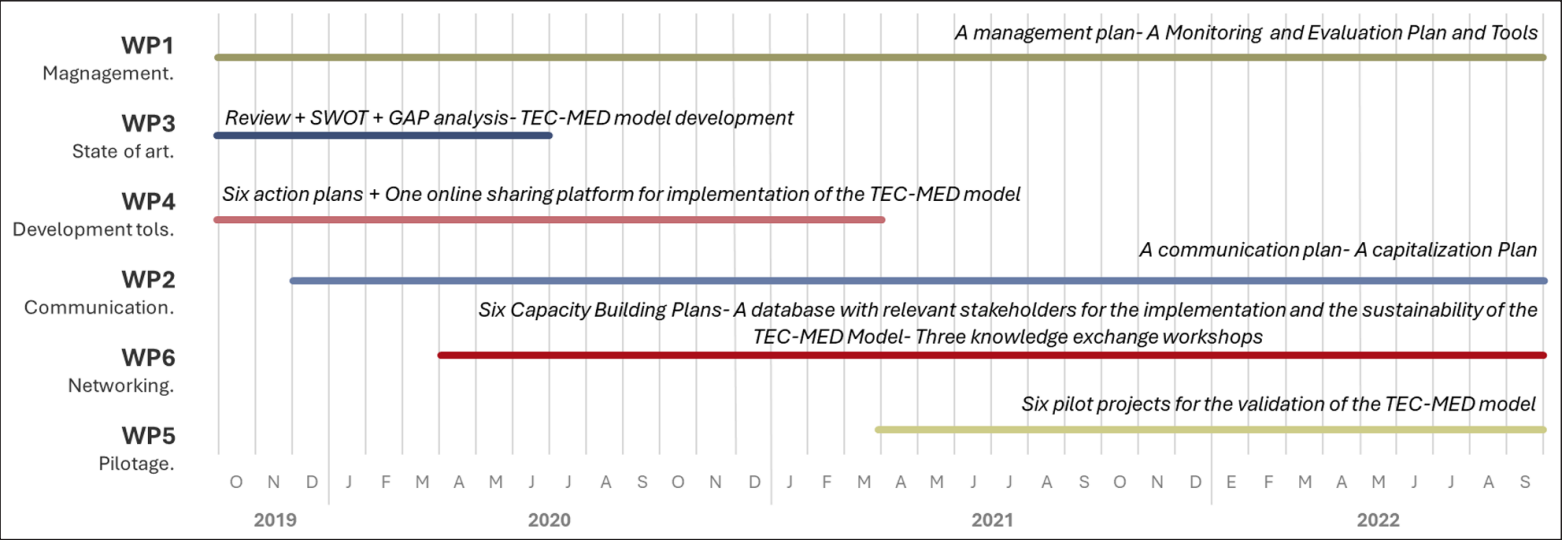
The schedule for the TEC-MED project.

If necessary, WP and activity leaders should schedule additional meetings, video calls, and teleconferences. WP leaders report regularly to the Project Management Team and the Steering Committee on the progress of their WP and deliverables. Figure 2 explains the chronological development of the main activities of each WP.

During the execution of the project, the main activities described below will be carried out:

- *Six pilot projects (detailed in WP 5)*. These will help provide evidence of the effectiveness and sustainability of the implementation of the TEC-MED intervention framework. The pilots are conducted in the six countries of the project, and in the course of their work, they will coordinate with NGOs and institutions acting as project partners, together with relevant local social care stakeholders.
- *Three knowledge exchange workshops (detailed in WP 6)*. Each international workshop is to be organized in one of the three cross-border areas of the project (that is, Italy, Greece, and Spain) to share experiences and know-how among NGOs, social care institutions, and relevant stakeholders. These allow institutional capacity building to provide social care services to the target population.
- *One online sharing platform (detailed in WP 4)*. This space will be used to facilitate communication and to leverage the organizational knowledge and capabilities of the participating organizations and stakeholders. Specific events are to be held within the platform, such as webinars and virtual demos.

The WP1 and the WP2 are related to management, while the rest, described below, are related to research. WP1.The project management team is made up of a project coordinator and a project assistant for the applicant and for each partner. WP 2. Focusses on creating awareness, engaging stakeholders, developing a communication plan, conducting networking activities, and effectively disseminating project results.

#### Work Package 3. State-of-the-Art: Analysis and definition of the Integrated TEC-MED Model (WP 3)

Based on the theoretical frameworks stated in the introduction, this WP is aimed to ultimate develop the TEC-MED Model. Social care professionals in each country will collaborate to develop the model for the target population in the six countries. First, a systematic review of the literature will be performed to identify the 20 most promising integrated social care initiatives in Europe and the Mediterranean countries. This review will include either implemented programs or theoretical models that exemplify the best practices and innovative aspects of social care for the target population.

Semi-structured interviews will be developed to analyze current social care practices in the six participating countries. In the interviews, the profiles described in the quadruple helix are considered as follows. Macro-management: leadership position in public administration that provides social-care services (may include policy makers and other stakeholders). Meso-management: leadership position of a social enterprise providing social care services, leadership position of a NGO providing social care services, leading academic figure studying or working in social-care services. Micro-management: social professionals who serve the dependent older people population with a risk of social exclusion (in a social enterprise, NGO, or similar) and represent the target population.

The last activity in this phase will be the diagnostic investigation of social-care practices in each country using SWOT and GAP analysis [19]. The problem that will be assessed is the current social care model in the country for the older people-dependent population and/or in the risk of social exclusion. It should be deepened in the characteristics of the model structure, including its principles/theoretical basis (including culture, elements of social inclusion or ethics, among others), legislation and norms, economic aspects or finances, target population (implementers and final population and other stakeholders), including educational aspects, strategies and tools, settings, and/or evaluation.

All these activities will help form the basis of the TEC-MED Model development, a model that uses an application of a conceptual new framework (care integrated into the person) to a new socio-health care model that covers the social determinants of health. A pioneering model that uses the integration of social and health aspects in dependent older people and at risk of social exclusion in the countries of the Mediterranean basin that have characteristics in common.

Coordinating country: Egypt, three activities and 12 months duration. All activities are described in Table 1.

**Table 1:** Activities included in Work Package three (WP3).


WP 3	STATE-OF-THE-ART: ANALYSIS AND DEFINITION OF CROSS-CULTURAL MODEL

Activity 3.1.	**Activity title:** Analysis of the most promising social care initiatives already existing in the Mediterranean basin **Activity Description:** Analysis of the most promising social care initiatives already existing in the Mediterranean basin and the EU and the selection of 15 case studies through an integrative review. Case studies will describe logic models, delivery, tools, governance, impacts, transferability, scalability, and include benchmarking analysis of key factors.

Activity 3.2.	**Activity title:** Analysis of current social care practices in the six countries involved in the project and SWOT Analysis. **Activity Description:** At least three semi-structured interviews are conducted with key stakeholders in the social care process in each country. These will be based on the same checklist as that developed for the identification of the most promising case studies in A3.1. The interviews will be analyzed using the SWOT analysis technique.

Activity 3.3.	**Activity title:** Gap Analysis and TEC-MED Intervention Framework Definition **Activity Description:** Workshops with stakeholders in each country will discuss key elements of initiatives to improve care models and refine the framework. Finally, with all the collected data the social care TEC-MED model for vulnerable older population will be developed by consensus of all the partners.


#### Work package 4. Development of a multicultural, solidarity-based social care model and tools (WP 4)

The objective of WP 4 is to develop 6 action plans, including the evaluation framework and the platform for the operationalization of the model. The starting points of the activity are the results of WP 3. APs include features of transcultural older people care, supporting collaboration between TAs, social care management teams from NGOs, patients, and their families.

In parallel, the relevant software is adapted to the local model of social care. The features of the online platform are mainly orientated towards the empowerment of the older people and toward ubiquitous communication and collaboration. The TEC-MED platform is codesigned in cooperation between public institutions and social-care organizations to be used anywhere and at any time in the specific region/towns. This will allow TAs, social care management teams from NGOs, patients, and their families to receive instructions, share best practices and experiences, and receive training and coaching support in a well-established community of practice.

For the development of the platform, an assessment will be included for the target population having as outcomes the level of dependency, family health, or the quality of life. The following assessment scales for users will be included: INICIARE [20], AROPE (At Risk of Poverty and/or Exclusion) [21], QoL (EQ-5D-5L) [22], Health Literacy (HLS-EU- Q16) [23], and Time Use Survey (TUS) [24]. For caregivers, the following scales have been included: INICIARE [20], QoL (EQ-5D-5L) [22], Health Literacy (HLS-EU- Q16) [23], Caregiver Burden Scale [25], Self-perception of Family Health Status [26], and Time Use Survey (TUS) [24]. Additionally, NANDA International Nursing Diagnoses [27], NIC (Nursing Intervention Classification) [28], and NOC (Nursing Outcomes Classification) [29] will be used. The nursing taxonomy will be adopted because it establishes, it offers a standardized language and is widely used and international in scope, which facilitates the homogeneity of the data and the application of the model. The information collected through the online platform will be useful for increasing the knowledge of model perception and to collect feedback from the ground in view of the next future implementation of a wider integrated social care model.

Coordinating country: Spain, two activities and 24 months’ duration. All activities are described in Table 2.

**Table 2:** Activities included in Work Package four (WP4).


WP 4	DEVELOPMENT OF A MULTICULTURAL, SOLIDARITY-BASED SOCIAL CARE MODEL AND TOOL

Activity 4.1.	**Activity title:** Operationalization of the Intervention Framework **Activity Description:** This activity elaborates the action plans for the partners’ institutions to develop strategies in collaboration with the public administrations in charge to support the older people who have chronic diseases and are at risk of exclusion. They include the provision of transcultural older people management features, supporting collaboration among TAs, social care management teams of NGOs, patients, and their families.

Activity 4.2.	**Activity title:** Development of a multipurpose online platform **Activity Description:** The online platform is designed for use by social care organizations, citizens, and public administrations for social-care model implementation and monitoring outcomes. It supports TAs, social care management teams of NGOs, patients, and their families in training activities, sharing experiences and best practices, receiving training and coaching support in a well-established community of practices. The online multicultural platform is enabled through software developed by PP1 and PP3.


#### Work package 5. Validation of the TEC-MED social care model (WP 5)

The project includes two levels of intervention. On the one hand, it includes cross-border staff exchange and joint training among social service people working for public authorities (training agents), allowing the exchange of experiences and best practices. On the other hand, on the nongovernmental level is based on working with associations, social care service entities, professionals, and technicians.

Coordinating country: Lebanon, two activities and 24 months’ duration. The activities of this WP are specified in Table 3.

**Table 3:** Activities included in Work Package five (WP5).


WP 5	VALIDATION OF THE TEC-MED SOCIAL CARE MODEL

Activity 5.1.	**Activity title:** Implementation of the TEC-MED model **Activity Description:** The model will be tested in different countries. Each partner identifies and trains the professional profiles of the training agents using information and professional experience, taking into account the sociocultural characteristics of their countries.

Activity 5.2.	**Activity title:** Validation of the TEC-MED model **Activity Description:** A quantitative (effectiveness and economic) and a qualitative evaluation will be done. The TEC-MED model will be modified according to the results obtained during the pilot (A5.1.2.) until the sample size is verified and its validation verified. The model will be certificated by a Certification Company. This activity also involves turning these professionals into trainers of future social care professionals and granting those tools and knowledge to increase the capacities and competencies related to the care of the older people population.


A total of 28,200 citizens (4,700 in each country) will be involved in the implementation phase; these will mainly be the older people targets of the TEC-MED social integrated care model. This target includes both older adults and their caregivers, whether they are living at home or in nursing homes. Each country can determine the proportion of the target population that is from homes and nursing homes, keeping in mind that caregivers are also a target population. (The general suggestion is to have 40% of the population from institutions and 60% from homes.)

First, a mapping of centers and institutions for the care of older people will be made through meetings with relevant stakeholders in the participating countries, to implement the TEC-MED care model, based on the number of residents and/or users they serve, the availability of infrastructure, geographic representation, and accessibility to them. Formal agreements will be made with the selected data collection centers.

Second, the identification and selection of training agents is performed to implement the TEC-MED model. Training workshops for the training agents on the TEC-MED care model, also training them to become trainers within their organizations.

Third, TAs are expected to visit the older people three times, including a first assessment, a three-month follow-up, and a six-month follow-up. Two groups are included: the experimental group, which will receive an intervention based on the TEC-MED model, and a control group from waiting lists.

In the intervention group, each TA is expected to spend between 30 minutes and 1 hour of social care intervention at home per patient every 3 months. These interventions will be adapted depending on the needs of the people being treated. They will be interventions related to healthy eating, mobilization and strengthening with physical exercise, coping with grief, among others.

**Figure 3:**
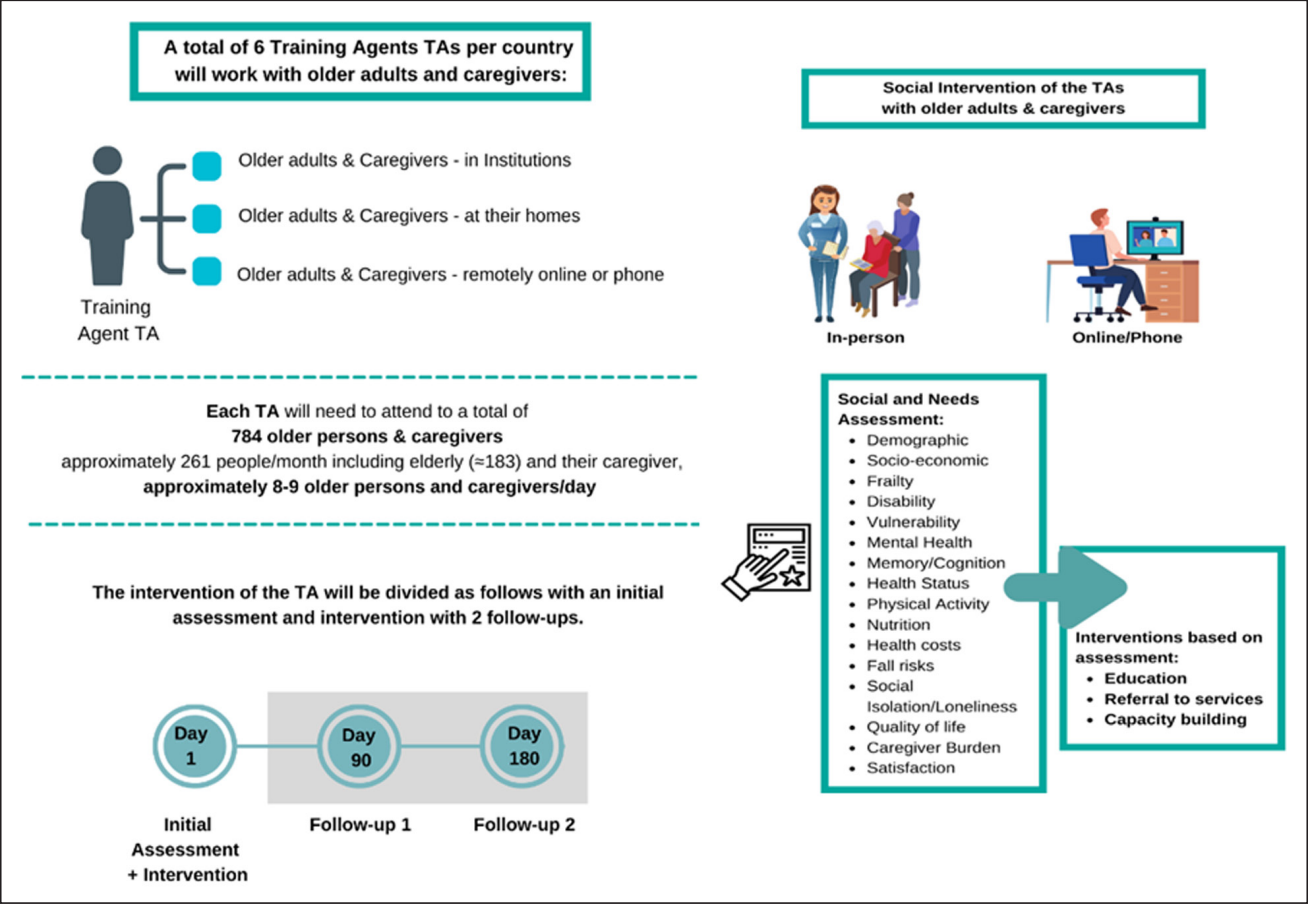
Targets for Training Agents and possible Interventions.

Furthermore, each TA will be expected to perform trainer training for future agents on the proper use of the platform and the TEC-MED model (Figure 3).

The intervention is projected to consist of two primary components: 1) Assessment component and 2) Intervention component.

The assessment component will involve screenings utilizing standardized scales incorporated into the platform and the following aspects: demographic, social isolation/loneliness, socio-economic and financial status, dependence, frailty, disability, vulnerability, mental health, memory/cognition, health status, physical activity, nutrition, health costs, fall risks, quality of life, caregiver burden and satisfaction.

The intervention component will be guided by the outcomes of the assessments. If any screenings yield positive results, an intervention will be initiated, and recommendations will be provided to address the identified issues. Interventions may include educational initiatives, referrals to services, engagement with relevant institutions, capacity building, among other strategies.

Each country has the flexibility to adapt the social care interventions, ensuring alignment with the established general framework and the overarching goal of reducing social isolation and exclusion.

At the end, the model will be modified according to the results obtained throughout the pilot until the sample size and validation are reached with all partners. Pre- and post-data will be collected for training, changes in scales, and assessments before and after the intervention. Moreover, qualitative analysis will be performed through in-depth interviews and focus groups to gain a greater understanding of the subject. In addition, validation activities will be conducted by a certification company through careful audits in which the state of implementation of the model is analyzed through the indicators developed.

Once the model is validated, future agents will begin to be trained, both direct practitioners and policy makers. Each project partner will identify suitable training agents based on the information provided and their professional experience, considering the socio-cultural characteristics of their respective countries. The project will end with an evaluation of the training.

#### Work package 6. Community building, stakeholder engagement, and capacity building (WP 6)

The outcome of this work package will be to identify and create a relevant community of stakeholders around the project and its themes. The intention here is to build a strong network of experts, practitioners, policy makers, civil society, organizations, and senior citizens across typologies and to have an involved community that remains involved in joint activities following the end of the project. Partners will build a strong network of stakeholders that will participate in networking activities such as the project mailing list to define its expansion strategy and the spread of excellence. Engaging stakeholders in the project will enhance outcomes, define the social care model for dependent and/or at-risk older adults, access beneficiaries, and explore ways to implement and disseminate the model.

This community participation will take place through four international workshops, where ideas will be exchanged, and stakeholders will be able to integrate stakeholders in decision -making

Coordinating country: Tunisia, three activities and 24 months’ duration. The activities of this WP are specified in Table 4.

**Table 4:** Activities included in Work Package six (WP6).


WP 6	COMMUNITY BUILDING, STAKEHOLDER ENGAGEMENT, AND CAPACITY BUILDING

Activity 6.1.	**Activity title:** Identified Stakeholders and Networking Activities Planning **Activity Description:** The main goal of this activity is to identify stakeholders and design a strategy to build a strong network of experts, practitioners, policy makers, civil society organizations, and senior citizens participating in the project.

Activity 6.2.	**Activity title:** Validation of the TEC-MED model **Activity Description:** In this task, a plan will be designed to involve stakeholders in the project. The plan will then set out the varying methods and engagement activities, including workshops, webinars, community collaboration portals, social media, the project homepage, brochures, stakeholder -specific briefs, and participation in relevant events to deliver an open, widespread, and self-standing network of stakeholders that will be the main audience of the project and will define its expansion strategy.

Activity 6.3.	**Activity title:** Validation of the TEC-MED model **Activity Description:** The project team will organize a set of periodic events to present the results of the project and to which relevant stakeholders will be invited to increase their competence and knowledge about the project theme. In addition to these events, webinars and online engagement in the best practice repository will stimulate capacity building.


### Data Analysis

Qualitative data from interviews and the focus groups will be recorded (only audio) for their transcription, categorization and analysis. They will translate into English to redact a summary of the main points of the discussion; the summary was shared with the partners afterward. A content analysis will be carried out to generate the most prevalent thematic categories [30]. The AtlasTi^©^ or NUDIST^©^ are used to support qualitative analysis. Data analysis is triangled by two members of the research team in each participant country. Later a member of the leader beneficiary will review them.

#### Quantitative data (effectiveness and economic evaluation)

After collecting data on the effectiveness evaluation of the implementation of the TEC-MED care model, the relevant descriptive and bivariate statistical analysis will be performed, studying the existence or lack of significant differences according to the comparison variable. Previously, the data will be subjected to the normality study, by means of the Kolmogorov-Smirnov test, which will determine the use of parametric or non-parametric statistics. Statistical significance will be established at a value of p > 0.05, and 95% confidence.

In addition, regression studies will be implemented to obtain predictive results, and odds ratio calculations, considering the longitudinal nature of the study.

Regarding the quantitative evaluation, the expected impacts of the project according to Annex 1 are: a) > 70% increase in patient’s activities of daily living b) health costs <= 5% reduction; c) 1–2 years increase in patient’s long-term quality-adjusted life years (QALYs); d) significant improvements in social inclusion of target patients. For data analysis SPSS V26 software will be used.

Regarding the economic evaluation, an evaluation of the cost-effectiveness and cost-utility of the TEC-MED model will be carried out from a societal perspective, using the incremental cost-effectiveness ratio (ICER) as a final measure.

The health outcome measure taken for the cost-effectiveness analysis will be the reduction of dependency in the older people and the reduction of social exclusion. (through the INICIARE-40 and AROPE questionnaire, respectively).

The health outcome measure taken for the cost-utility analysis will be the quality-adjusted life years (QALYs) calculated from survival and the utility indices of the generic questionnaire that measures quality of life of the EuroQoL group known as EQ- 5D-5L [31,32,33,34,35]. To determine the cost-utility, we use the willingness-to-pay threshold for participant countries. The uncertainty will be measured through deterministic and probabilistic sensitivity analyses.

### Ethical considerations

Prior to their involvement, all participants provided written informed consent, acknowledging their voluntary participation. It was made explicitly clear to participants that they retained the right to withdraw from the discussions at any point, without any repercussions. To protect their privacy, all audio recordings of the interviews were securely deleted once the accuracy of the transcripts was verified.

Importantly, this study was conducted as part of the European TEC-MED Project (A_A.3.2_0376) research project of ENI CBC 2014-2020, and permission was obtained from the Andalusian Research Ethics Committee (2412-N-19).

## Discussion

This study develops an innovative integrated transcultural social care model to improve the daily operations of social enterprises and the collaboration with the public administration in the care of older people with dependency and at risk of exclusion. At this time, the integrated care model has been used in different populations, thus improving their care and attention. For example, an integrated care systems model was implemented in cancer patients, demonstrating numerous benefits in the care experience and clinical outcomes [36]. Similarly, this model has also been applied to chronic patients, showing how the use of multidisciplinary care better adapted to the specific needs of patients in terms of safety and patient-centered care [37].

Finally, the use of an integrated care model has shown improvements in a population similar to the one included in this study, older people living at home, where hospital readmissions were reduced, and there was an improvement in the symptoms presented [38]. Therefore, the integrated social care model could achieve these positive results for older people with dependency and/or the risk of social exclusion living in the Mediterranean basin. These positive benefits can translate into an improvement in quality of life, since there is evidence that relates social health care with improved quality of life in patients with heart problems and in older people people with complex needs [39,40].

Currently, some Mediterranean countries are more advanced in the ageing transition, while in others, the process is underway with acceleration due to the migration phenomena of younger generations [12]. All showed a common pattern of decreasing social support and a need for innovative and sustainable social care models [41]. This has led to significant repercussions for many categories of vulnerable people, including the targets of the TEC-MED project, such as dependent older people with chronic diseases and lack of support from the family network highly affected by the lack of policy priorities and new welfare systems models, which further marginalize the scarcely developed social assistance systems [14,42].

All countries and regions surrounding the Mediterranean Sea basin have been showing an increase in the share of older citizens in the active population. More specifically, all Mediterranean countries, albeit to different extents, are experiencing an increase in the life expectancy of citizens and, therefore, in the aging index and in the older people dependency ratio, resulting in a greater need for public spending in social services to meet higher social demand and sustain social cohesion [43,44].

Unfortunately, the public resources that support these services are scarce. In fact, the northern shore of the basin has been hit by a banking crisis stemming from losses in capital market securities driven by the spread of new financial instruments and derivatives, as well as a sovereign debt crisis exacerbated by recession. These crises have led to a general downturn on the southern coast due to weak export markets in Europe due to the recession caused by high international oil and food prices, and a drop in tourism receipts, which in 2010 accounted for more than 20% of GDP in Lebanon and between 5% and 8% in Tunisia and Egypt [45]. At the same time, the project focuses on addressing the financial challenges created by current account deficits and increased debt levels, while also emphasizing social inclusion, poverty reduction, and support for social and solidarity economic actors. Special attention is given to providing care to dependent older people individuals with chronic diseases who lack family support, and all project initiatives will be rigorously tested in various cross-border areas and with different care professionals and organizations to ensure their effectiveness and relevance. Concerning the reduction in healthcare costs, an economic evaluation of the activities conducted will be performed to confirm the savings in the social and healthcare systems following the model’s validation [31,32]. The validation activity of the TEC-MED model pilots includes the participation of relevant stakeholders from the six countries involved.

The main limitations of the study will be related to the COVID-19 crisis [46]. This crisis could delay the training of the TA and the initiation of the pilot phase. In addition, the accessibility of the target group could be more difficult because most are an at-risk and vulnerable population. The results will be transferable to all social care organizations in the areas impacted by the policies. Transferability barriers and regulatory constraints related to setup costs and usability are not expected to hinder project results.

## Conclusions

The project expects to include a TEC-MED integrated social care organizational model including a governance model, an organization structure, and skills profiles for the social care personnel. It will provide a software solution to enable an organizational model of social care, including specifications for its localization and deployment in reference countries. A set of action plans will be produced to improve and adapt social policies to be implemented in cooperation with public administration and including other sectors, such as NGOs and private centers in Mediterranean countries. In conclusion, 28,200 people will be covered by improved social services, 12 agreements will be concluded between public administrations and relevant stakeholders for the coordinated planning and implementation of social services, six action plans will be droughted for public administration, and one online platform will be created for cooperation and partnership between public institutions and social care actors. Finally, a new social-ethical care model will be validated in the target population and adapted in different countries with singular social differences.
